# Global concerns of dental and oral health workers during COVID-19 outbreak: a scope study on the concerns and the coping strategies

**DOI:** 10.1186/s13643-020-01574-5

**Published:** 2021-02-02

**Authors:** Peivand Bastani, Mohammadtaghi Mohammadpour, Arash Ghanbarzadegan, Kostas Kapellas, Loc Giang Do

**Affiliations:** 1 Health Human Recourses Research Centre, School of Health Management and Medical Informatics, Shiraz University of Medical Sciences, Shiraz, Iran; 2 Student Research Committee, Shiraz University of Medical Sciences, Shiraz, Iran; 3 School of Health Management and Medical Informatics, Shiraz University of Medical Sciences, Shiraz, Iran; 4 Australian Research Centre for Population Oral Health (ARCPOH), Adelaide Dental School, University of Adelaide, Adelaide, South Australia Australia

**Keywords:** COVID-19, Oral health, Dental health, Global concern, Coping strategy

## Abstract

**Background:**

Dental and oral health workers have direct contact with respiratory aerosols of patients during procedures. This study aimed to determine the main concerns of dental and oral health workers globally during COVID-19 outbreaks and the coping strategies that help the resilience of dental and oral healthcare system.

**Methods:**

This scoping study was conducted in August 2020. After adjusting the search strategy, a systematic search of five databases (PubMed, ISI Web of Science, Scopus, ProQuest and EMBASE) was conducted. Data was extracted using Microsoft Excel and the contents of retrieved articles were analysed through a qualitative thematic analysis applying MAX QDA_10_.

**Results:**

Most articles were either editorial/letters to the editor/commentary formats (34%), or literature reviews (26%). About half of the articles belonged to three countries of Italy, China and the USA (each 16% and totally 48%). Thematic analysis of included papers led to the identification of four main global concerns and 19 sub-concerns. Economic, ethical, social and professional concerns are among dental and oral health concerns. Other results indicate on three main themes and 13 sub-themes as the coping strategies including patient management, infection control and virtual strategies.

**Conclusion:**

Dental and oral health care workers have many concerns relating to COVID-19 including economic, ethical, social and professional factors. Resolution of concerns may involve enhancing coping strategies relating to patient management and infection control strategies as well as using new technologies for virtual contact with the patient without any risk of infection.

**Supplementary Information:**

The online version contains supplementary material available at 10.1186/s13643-020-01574-5.

## Background

COVID-19 is the latest respiratory contagious disease that has spread rapidly around the world [1]. It was first discovered in December 2019 in Wuhan, China, but quickly spread across the globe to the point that in March 2020, the World Health Organization (WHO) declared it a pandemic and a public health emergency of International Concern (PHEIC). In the United States of America (USA), Spain, Iran and Italy, the disease has drastically progressed over time [2].

COVID-19 has a high rate of human-to-human transmission through respiratory aerosols which remain stable at various levels for a long time. Recommended preventive measures including staying at home, implementing travel bans, maintaining social distancing, frequent hand washing and using personal protective equipment (PPE) such as masks and gloves can minimize the risk of transmission and infection [3]. The greatest risk of infection is among healthcare workers who are at the frontline and in direct contact with patients. Consequently, many have contracted COVID-19 and some lost their lives [4].

The New York Times, published an article titled “*The Workers* Who Face the Greatest Coronavirus Risk” explained that dentists are more at risk than general practitioners and nurses in getting COVID-19 [5]. Dentists and other dental and oral health workers are allied members of the frontline healthcare workforce at extreme risk of COVID-19 infection due to generation of aerosols as part of providing dental care [6]. During previous pandemics, spreads of contagious respiratory diseases such as SARS and the Middle East respiratory syndrome (MERS) were contained following development of protective protocols and enhanced infection control guidelines that enabled provision of dental services in a safe manner [7, 8]. With the COVID-19 pandemic, various dental associations have taken important steps against the disease. The American Dental Association has set up various ways to answer dentists’ questions about personal protection and safe practice, for example, the recommendation that dentists use PPE and evacuate patients’ saliva using a high-volume suction device to minimize aerosol production [9].

Providing clear and convenient guidelines for managing dental patients by reputable journals and dental associations is essential to ensure lowering risk from dental procedures. A basic concept is that the transmission of the virus occurs mainly through inhalation, eating and drinking and direct mucosal contact with salivary droplets. The virus can also survive on dental equipment for up to 9 days [10]. Because viral load in human saliva is high, the use of mouthwash can only reduce the microbial load in the mouth and does not have the potency to completely eliminate COVID-19 [7, 11]. Therefore, most guidelines recommend that dentists should not accept a new patient in the current situation, sparing an emergency. This significantly reduces interpersonal contact and patient waiting times and minimizes exposure of patients to the infection. Also, dentists should check patients’ fever and ask questions about the patient’s general health before attempting any treatment [12–14].

The effects of this crisis on dental services, such as the restriction of dental practices to emergencies, the shutdown of many dental centres and the risks of infection transmission, can be major concerns of dental care providers [15]. Therefore, the aim of this study was to investigate the literature related to the concerns of the dental and oral health workers in the COVID-19 pandemic in order to identify the most frequently raised issues and the suggested coping strategies. This scoping review aims to provide evidence for dental and oral healthcare workers to enable a better understanding of these concerns. Additionally, this review may aid policy makers to make better decisions and increase the resilience of dental and oral health system against COVID-19 outbreak.

## Methods

This scoping review was conducted in 2020. Scoping reviews are usually applied to map the key concepts based on the research area as well as clarifying definitions, determinants and the conceptual boundaries of a topic [16]. In this regard, Arksey and O’Malley [16] proposed a framework with six consequential steps to run a scoping review. This framework has become updated by Levac and O’Brien [17] and Peters et al. [18] with 6 and 9 steps respectively. In this study, the framework of Arksey and O’Malley was applied. This framework contains 5 mandatory steps and one optional step including the following: (1) identifying the research questions, (2) identifying the relevant studies, (3) study selection, (4) charting the data, (5) collating and (6) summarizing and reporting the results and consultation (optional) [16]. For more clarification of the present method, each of the five steps as follows are described:

### Identifying the research question(s)

The following questions were explored:

1. “What are the main concerns of dental and oral health workers during the COVID-19 outbreak?”
2. “What coping strategies can be applied by oral and dental health care workers to have a more resilient system during and post COVID-19 outbreak?”

### Identifying the relevant studies

For this purpose, the literature search was conducted in five databases including PubMed, ISI Web of Science, Scopus, ProQuest and EMBASE by applying related and appropriate keywords. Logical operators OR /AND were used to combine keywords and increase the sensitivity of the search. The search strategy presented in Table 1 sourced articles published up to 21.08. 2020. Google Scholar was additionally searched via the research topics. These databases were selected because they cover most of the fields in medicine, public health and dental and oral health.

**Table 1:** The search strategy of the scoping review

**Web of Science**	TOPIC: (COVID-19) OR TOPIC: (“CORONA virus”) OR TOPIC: (SARS) OR TOPIC: (MERS) OR AND TOPIC: (Dentists) OR TOPIC: (“Oral Health”) OR TOPIC: (“Dental Technicians”) OR TOPIC: (“Dental Care”)
**Scopus**	TITLE-ABS-KEY (dentists) OR TITLE-ABS-KEY (“Oral Health”) OR TITLE-ABS-KEY (“Dental Technicians”) OR TITLE-ABS-KEY (“Dental Care”) AND TITLE-ABS-KEY (“COVID-19”) OR TITLE-ABS-KEY (“CORONA virus”) OR (“H1N1”) OR TITLE-ABS-KEY (“outbreaks”) OR TITLE-ABS-KEY (“epidemic”) OR TITLE-ABS-KEY (“pandemic”)
**ProQuest**	ab(COVID-19) OR ab(“corona virus”) OR ab(SARS) OR ab(h1n1) OR ab(outbreaks) OR ab(epidemic) OR ab(pandemic) AND ab(Dentists) OR ab(“Oral Health”) OR ab(“dental technicians”) OR ab(“Dental Care”)
**PubMed**	((((((“COVID-19” [Supplementary Concept]) OR “Disease Outbreaks”[Mesh]) OR “Coronavirus Infections”[Mesh]) OR “Middle East Respiratory Syndrome Coronavirus”[Mesh]) OR “SARS Virus”[Mesh] OR “Influenza, Human”[Mesh]))) AND (((((“Dentists”[Mesh]) OR “Dental Care”[Mesh]) OR “Dental Health Services”[Mesh]) OR “Dental Technicians”[Mesh]) OR “Oral Health”[Mesh] OR “Specialties, Dental”[Mesh]) OR “Dental Hygienists”[Mesh]) OR “Dental Prosthesis”[Mesh])
**EMBASE**	#3 #1 AND #2 #2. dentist:ab,ti OR “oral health”:ab,ti OR “dental technician”:ab,ti OR “dental hygienist”:ab,ti OR dentistry:ab,ti #1. “coronavirus disease 2019”:ab,ti OR “middle east respiratory syndrome”:ab,ti OR “sars coronavirus”:ab,ti OR influenza:ab,ti

### Study selection

**Fig. 1:**
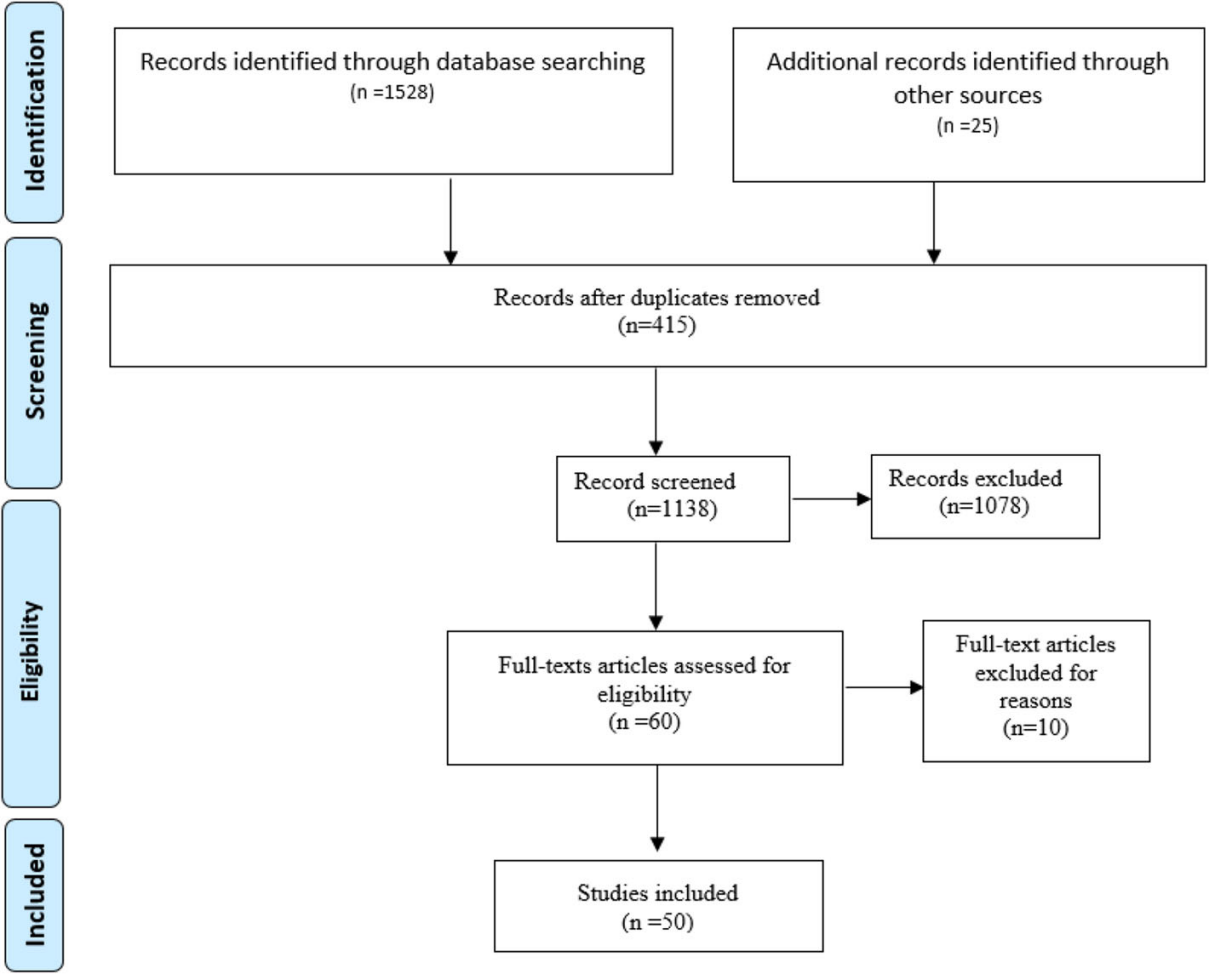
The scope study PRISMA flow chart

Following the pre-stated search strategy, all databases were searched and results were screened in three steps. First, titles, then abstracts were screened and finally full texts of relevant papers were downloaded. Articles not in English and references relating to conference proceedings were excluded. All scientific peer-reviewed publications in the form of original articles, reviews, commentaries and editorials with a full text were subsequently included (Fig. 1). Endnote X7.1, by Thomson Reuters, was used for managing the process.

### Charting the data

**Fig. 2:**
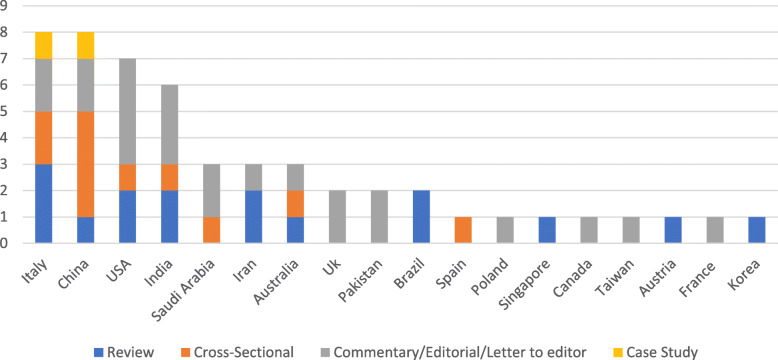
The status of included articles according to their place and design

Data extraction was performed using Microsoft Excel software. Author information, study location, year of publication, article title along with the aim and study design main results and conclusions were collected. Results are presented in Fig. 2.

### Collating, summarizing and reporting results

Extracted data was analysed through a qualitative thematic analysis via six steps [19]: (1) data familiarization occurred via multiple readings of the papers’ full texts; (2) initial open codes were identified based on the research question; (3) codes were reviewed, categorized and finalized; (4) revision and categorization of the final themes to develop main themes and sub-themes; (5) the main themes and sub-themes were revised through an extensive review to combine, refine, separate or discard initial themes wherever necessary; and (6) finally, the themes were defined, labelled and tabulated to report the final results applying the qualitative software MAX QDA_10_. A conceptual map presented as a thematic network in order to better understand the concept. All these analyses were conducted by two researchers (*PB and MM*) who have enough reflexivity in qualitative studies and scoping reviews with no conflict of interests for data analysis.

## Results

As the PRISMA flowchart presented (Fig. 1), among 1553 initial retrieved studies, 50 articles were included in the scoping review. A summary of the characteristics of included studies is presented according to the place of the study and the study design (Fig. 2). Most of the 50 included studies were in the format of editorial/letter to editor and commentary (17 articles, 34%) and 13 were reviews (26%). Further, 23 of 50 articles were conducted in Asian countries (46%), whilst 13 studies were from Europe (26%). Among those studies, Italy and China were the countries that have the highest proportions respectively. Other results according to the proportion of publication demonstrate that about half of the included articles (48%) belonged to three countries China, Italy and the USA (Fig. 2).

Thematic analysis of the results of the manuscripts is shown in Tables 2 and 3. Four main themes are achieved as the main global concerns of oral and dental health workers during COVID-19 outbreak (Table 2). These main concerns include economic, ethical, social and professional concerns that are followed by 19 sub-concerns (Table 2).

**Table 2:** Main concerns and sub concerns of dental and oral health workers

Main themes	Sub-themes	References
**Economic concerns**	Economic losses/financial consequences	[20–24]
	Economic repercussions	[20]
	Economic aid from the government	[20, 25]
	Additional costs of control infection	[20, 22, 26]
**Ethical concerns**	Patient privacy	[27]
	Effectiveness of new technologies on population health	[27–30]
	Focus on essential and emergency cares	[23, 24, 26, 27, 31–34]
	Postpone routine orthodontic appointments	[27]
	Concerns on fitness with international regulations	[28]
**Social concerns**	Lack of population’s adequate digital literacy	[28]
	High perceived distress and need for preparedness	[35, 36]
	Feelings of anxiety and fear	[32, 37]
	Psychological health and mental wellness of the workers	[33]
**Professional concerns**	Impacts of the future of professional	[25, 38]
	Immediate impact on dental health care	[36, 38]
	Impacts of management of patients with special needs	[39, 40]
	Medico-legal concerns	[36]
	various hardships in rebuilding the practices	[41]
	moral versus evidence based decision making	[22]

**Table 3:** Main coping strategies for oral and dental health workers against COVID-19

Main themes	Sub themes	References
**Patient management strategies**	Guidelines for patient management	[21, 26, 32, 33, 42–44]
	Training and education	[6, 26, 33, 39]
	Appropriate time management	[27, 30, 45]
	Patient screening	[6, 29, 46–49]
**Infection control strategies**	Personal protective equipment	[10, 12, 20, 22, 26, 27, 33, 44, 46, 48–52]
	Environmental sterilization and disinfection	[34, 42, 49, 52]
	Rubber dam isolation	[10, 12, 45, 51]
	Hand hygiene	[10, 12, 44, 48]
	Mouth rinsing and saliva ejection	[43, 44, 53]
	Removal of medical/dental waste	[10, 44, 48, 53]
**Virtual strategies**	Virtual assistant	[24, 31, 52]
	Telemedicine	[29, 30, 50]
	Tele dentistry	[31, 46]

### Economic concerns

The first global concern of oral and dental health workers in the included studies related to economic factors. These included economic losses due to the reduced number of elective patients [20], financial consequences following reduction of revenue and the increased costs of acquiring PPE and management of environmental disinfection [20, 26]. It is practically impossible to perform many routine dental procedures during the outbreak, so many dental clinics temporarily closed or only permitted limited emergency services leading to serious financial problems. In such complicated situations, the oral and dental health workers may hope for achieving economic support from the governments to help continuing dental procedures during COVID-19 outbreak [25].

### Ethical concerns

According to the present results, there are many ethical concerns for oral and dental health workers during the COVID-19 outbreak. The main repeated sub-theme was the obligation for restricting dental health services to the emergency conditions at the expense of preventive procedures. The emergency conditions may include those cases experiencing severe pain and discomfort [27, 37] or those who need hospital-based services [15, 21]. Other concerns are related to new issues such as the effectiveness of tele-dentistry [28–31] and lack of patients’ privacy via the tele-consultation methods [28]. Lack of potentialities for the best matching with the international regulations is among other ethical concerns [28].

### Social concerns

Social concerns encompass the psychological concerns of oral and dental workers akin to feelings of anxiety and fear of becoming sick [28, 32]. High perceived stress or distress among dental workers is thought to negatively impact on the preparedness against a COVID outbreak [35, 36], affecting the psychological, mental health and wellbeing of these workers [33]. Another aspect of social concerns relates to social responsibility of oral and dental health workers to assist members of the public who may lack sufficient digital literacy and are elderly, or those from rural, remote or otherwise deprived areas [28].

### Professional concerns

The last main concern achieved from the present results related to the dental and oral health professional rules. This concern both include the immediate impacts of COVID-19 on oral and dental health profession [36, 38] and the future of this profession [25, 38]. Many factors can affect the future of the oral and dental health profession including application of tele-health, tele-dentistry consulting and other new technologies that may find the opportunity to be used by dentists.

Among other professional concerns, managing patients with special needs must be considered [39, 40]. Patients, children and adults, with intellectual and learning disabilities may have little cooperation necessitating extended visiting times. This contravenes the requirement to minimize patient contact in line with COVID recommendations/guidelines and may cause some types of hardship in rebuilding the practices [41].

Table 3 presents three main themes and 13 sub-themes surrounding coping strategies that can help the oral and dental health system to preserve their resilience against COVID-19 conditions.

### Patient management strategies

Most included papers emphasized some form of guidelines or protocols for patient management be it professional judgement [32, 42, 54, 55] or the safety of dental workers [43]. Training and education was among other sub-themes of the patient management strategies. Two aspects of dental and oral health training and enabling them for the COVID-19 condition and after that [26] as well as the patient education are considered. In one online publication, case studies and problem-based learning were proposed as a strategy for education and training to prevent COVID infection and transmission [55]. At the same time, online training was recommended to teach patients how to deal with non-acute oral diseases in order to decrease the load of referrals to the dentistry centres [50].

Patient screening for COVID-19 status before admission for detecting the symptoms attributable to the disease is among another sub-theme [42]. This is important because due to the asymptomatic incubation period, there is a risk of transmitting the disease whilst providing dental services [22]. It is recommended that dental clinics have a pre-examination triage for patients visiting dental clinics. Additionally, it is best to have these rooms equipped with PPE. Fever assessment, history of contact with sick people, and history of travel to high-risk areas should also be considered [56].

### Infection control strategies

Infection control strategies included applying PPE, environmental sterilization and disinfection, rubber dam isolation, hand hygiene, mouth rinsing and saliva ejection as well as correct disposal of medical/dental waste.

Due to the special conditions of dental services, infection control is always one of the main concerns of dentists. One of the effective policies in this field is hand hygiene and disinfecting of surfaces. Coronaviruses can survive from a few hours to a few days depending on the type of surface, temperature, or humidity [57].

Given that the main mode of transmitting coronavirus is through respiratory droplets, use of standard PPE such as gloves, face shields and masks is very important whilst providing dental services. However, past experience with other respiratory illnesses suggests that dental procedures and treatments that reduce the production of aerosols must be applied to reduce risk of respiratory droplets [58].

Past experience shows that using mouthwash during previous SARS and MERS pandemics was effective in reducing viral load of saliva and reducing the ratio of microorganisms in aerosol particles [46, 59]. Precautions such as the use of disposable appliances such as syringes, blood pressure cuffs and mouth mirrors; reduced use of hand pieces and ultrasonic; and the use of extra-oral radiographs have also been recommended for greater safety and prevention of virus spreading [10, 12]. It is also important to use the waiting room with the proper ventilation system and the negative pressure isolation room to provide emergency services to patients.

One of the sub-themes was the practical waste management strategies in medical and dental centres. It is recommended to follow the waste management regulations in the waste disposal and also wastes which are related to suspected patients should be packed and disposed in non-leakable double-layered nylons and the involved environment should be thoroughly cleaned with disinfectant solutions [10].

### Virtual strategies

Virtual assistant, telemedicine and tele-dentistry are among the main sub-themes related to this strategy. Online counselling or applying social network applications are suggested for orthodontic emergencies. It was suggested to preferably avoid face-to-face visits wherever applicable for all patients with both fixed and removable orthodontic appliances [21]. Online counselling for non-emergency services can be an effective coping strategy during covid-19 condition. One study that looked at the state of dental hospitals in China found that all hospitals suspended non-emergency dental services, and more than 90% of them set up an online dental counselling system, which seems an effective policy during the outbreak of respiratory diseases [15]. Online video consultation for patients and online training of the public can be effective in this area [50]. Another included study emphasized applying tele-dentistry via phone calls or text messages as a very promising strategy to maintain contact with patients without risk of infection [31].

## Discussion

Results show that dentists and other oral health workers have experienced different concerns during COVID-19 outbreaks. These concerns are more common in four areas namely, ethical, economic, social and professional concerns. At the same time, other results indicate that applying the strategies of patient management, infection control and virtual approaches can fade these concerns and help the oral and dental health systems to preserve and increase their resilience.

The present results suggest emergency and non-emergency dental services, age-group services, orthodontic emergency tele-screening, training and online professional consultation are all avenues to maintain dental services in the presence of COVID-19 pandemic. The usefulness of tele-dentistry may be particularly important for oral health policymakers [23, 34, 60]. Lancheros-Cuesta et al. [47] emphasized that the use of information technology (IT) and telecommunication can be effective in the detection, prevention, treatment and control of dental problems. Similarly, strict and effective infection control protocols are strongly recommended for dental emergency practices in infected regions [6]. Applied training of oral health workers particularly for patient screening, infection control strategies and patient management can be very beneficial [46]. These strategies along with allocating separate centres for emergency and non-emergency practices during the COVID-19 pandemic and high access to protective medical equipment for oral health workers and the patients can greatly decrease the dentists’ concerns.

At the same time, other results have focused on local guidelines, dental education, occupational health, ethical issues and financial problems. In this regard, Coulthard [22] confirmed that although dentists have the moral and legal duty to reduce their routine care for preventing the spread of the COVID-19 among their patients and clients, they may encounter the fear of financial problems. It is obvious that in such a situation, it would be governments’ and policymakers’ task to assess potential financial losses and to find alternatives for compensating and allaying concerns. Other policymaking and management interventions can be concentrated on regulating new occupational standards or revising and moderating the previous ones. Such standards can assure the oral health workers access an optimal space for service delivery with lower risk of the disease [5]. At the same time, enough attention and closed supervision on implementing applied local guidelines can reduce the dentists’ concerns. These guidelines should contain the standard precautions for daily practice and also the special precautions [12] according to the intensity of infection and the local condition.

Other practical strategies during dental treatment consist of patient evaluation, hand hygiene, personal protection, mouthwash use, surface disinfection and waste management. In this regard, it is emphasized that COVID-19 may become airborne through aerosols formed during dental procedures [61]. Therefore, dentists must pay much more attention to the infection control principles as well as appropriate and adequate use of PPE for the workers’ health, and disposable appliances for the patients’ protection. The protective equipment includes diverse types of protective goggles and face shields, face masks, protective outerwear and gloves [44]. At the same time, before applying any strategies, dentists should be able to evaluate the patients for probable suspected cases [53]. It is obvious that the dentists should be aware of the symptoms of the disease and prevent treating the suspected cases and refer them to the related centres. Hand hygiene is another strategy that should be noticed by the dentists in two ways of continuous handwashing with water and soap and hand disinfection applying alcohol-based solutions [44]. Surface disinfection and waste management are among the practical strategies. Disposable and consumable equipment should be taken to a temporary storage area. It is important to note that, those of suspected cases to COVID-19 should be considered as infectious wastes [51].

Finally, according to the results, it seems that concerns caused by COVID-19 can highly affect the dentists’ service delivery. These proposed strategies can help the oral and dental health workers move toward different approaches during and post COVID-19. These new approaches can vary from optimizing the oral and dental health care standards to enabling positive attitudes and introducing constructive changes in oral and dental health workers [39].

## Conclusion

COVID-19 has caused many concerns for dental and oral health care workers including economic, ethical, social and professional concerns. These concerns can be alleviated by applying certain coping strategies such as patient management and infection control strategies as well as using new technologies for virtual contact with the patient. It would be obvious that in a case of appropriate policymaking and management, most of the oral and dental health workers’ concerns may be addressed and the oral health system may become resilient. It is important to remember that although the pre-stated concerns can be mentioned globally, each country may apply diverse local strategies to improve the oral health system’s resilience. All these strategies are recommended to be used as policy learned or lessons learned for future policymaking.

## Limitations

This scoping study has applied to achieve the main global concerns of oral and dental health workers and the coping strategies to help oral and dental health preserve its resilience. These coping strategies need to be tested in a different setting based on the technological, managerial and social context.

## Supplementary Information

**Additional file 1.** Supplementary tables

**Additional file 2.** PRISMA-ScR Checklist

## Data Availability

Whilst identifying/confidential patient data should not be published within the manuscript, the datasets used and/or analysed during the current study are available from the corresponding author on reasonable request.

## Abbreviations

MERS: Middle East respiratory syndrome
PRISMA: Preferred Reporting Items for Systematic Reviews and Meta-Analyses
WHO: World Health Organization
PHEIC: Public Health Emergency of International Concern
PPE: Personal protective equipment
