# Supplementary material for: Global concerns of dental and oral health workers during COVID-19 outbreak: a scope study on the concerns and the coping strategies
Source: Syst Rev. 2021 Feb 2;10:45. doi: 10.1186/s13643-020-01574-5 (PMC7850712; doi:10.1186/s13643-020-01574-5)
Supplement: Supplementary file 1 — Additional file 1. Supplementary tables [file 13643_2020_1574_MOESM1_ESM.docx]

Appendix – The main characteristics of the studies

| **No** | **First author** | **Place** | **Study design** | **Main directions** |
| --- | --- | --- | --- | --- |
| 1 | Parin Bhanushali | India | Review article | infection control strategies and patient management protocols for waiting rooms, during treatment and post treatment |
| 2 | Anil Kumar Ramachandran Nair | India | Online survey | High perceived distress among endodontists than the public, females and younger aged have higher risk for (dis)stress. monitoring, screening, referral and targeted intervention can decrease it. |
| 3 | Muhammad Adeel Ahmed | Saudi Arabia | Cross-sectional | Despite having a high standard of knowledge and practice,  dental practitioners around the globe are in a state of anxiety and fear while working in their respective fields due to the COVID-19, recommended guidelines to emergency treatment only or closed down practices for an uncertain period. |
| 4 | Kamran Ali | UK | Commentary | focus on emergency and preventive dental care, follow the most updated national and international guidelines, Additional costs related to PPE, staff training, and the investment to improve clinical environment, explore appropriate platforms to actively participate in research aimed at informing evidence-based protocols |
| 5 | H. Bahramian | Iran | Perspective | obliged to manage emergency situations such as cellulitis, severe tooth pain,  and dental trauma., beware of the symptoms and risks of the emerging disease, change the policies in our offices to minimize the risk of transmission |
| 6 | Ida Barca | Italy | Case study | management of maxillofacial patients, Preoperative treatment, Intraoperative protection, Postoperative management, |
| 7 | Alberto Caprioglio | USA | Editorial | Personal protective equipment, filter of patients who come to the ambulatory, postpone routine orthodontic appointments (except emergencies experiencing pain or discomfort), providing appropriate timely management, Virtual assistance |
| 8 | Roberto Careddu | Italy | Mini review | Infection Control, Patient Screening, , Patient Management, Air Supply, disinfection, |
| 9 | Cintia Chamorro-Petronacci | Spain | Descriptive Observational | Economic losses and repercussions, personal protective Equipment, only emergency care was available, providing special economic aid |
| 10 | Nicola Cirillo | Australia | Letter to editor | Transmission routes, Guidelines for dental and oral health Practitioners, Preparedness plan and streamlining of dental Procedures, Early recognition and prevention of transmission before dental treatment, Environmental cleaning and disinfection, Infection control precautions and practices for oral healthcare workers |
| 11 | Ugo Consolo | Italy | Online survey | COVID-19 emergency is having a highly negative impact on the activity of dentists practicing (feeling of concerns, anxiety, fear), concerns about professional future and the hope for economic measures to help dental practitioners |
| 12 | Kashif Naqvi | Pakistan | Commentary | Pre-procedural mouthwash, Use of rubber dam, Use of high volume evacuator, HEPA filters, Prioritize urgent care, Screening for COVID-19 status, Waiting room and arrival protocols |
| 13 | Arkadiusz Dziedzic | Poland | Editorial | management of patients with special needs |
| 14 | Fazal Ghani | Pakistan | View point | Immediate impact on dental health care, heavier impacts on the profession of dentistry |
| 15 | Amerigo Giudice | Italy | Literature search | Transmission routes, infection control in the dental office, patient management, prevention of cross infection in clinical & nonclinical areas, patient evaluation, telemedicine |
| 16 | Jincai Guo | China | case report | Effective measures: build a team of experts to advise patients with oral disease, online video consultation for patients, online training to teach patients how to deal with non-acute oral diseases, educate the public |
| 17 | Ana Luiza Barbosa JUREMA | Brazil | literature review | Personal Protective Equipment, Rubber dam isolation, High-speed handpiece, Disinfection and sterilization procedures |
| 18 | Niraj Kinariwala | India | Short communication | The perceived preparedness and anxieties of dentists in the provision of patient care, personal protective equipment, pandemic has led to an array of medico-legal and professional concerns for Indian dentists in terms of patient care provisions., |
| 19 | Parthasarathy Madurantakam | USA | Commentary | guidelines are followed given that most elective dental procedures generate high amounts of aerosols and a significant number of patients could be asymptomatic carriers |
| 20 | D. Maret | NA | Letter to the editor | Integration of telemedicine into the public health response and oral health |
| 21 | Marco Mascitti | Italy | Editorial | The transformation of DPH, a number of economic, ethical, and social concerns (real impact and effectiveness of new technologies on population health) (the lack of  adequate digital literacy among those groups that would benefit most from public health programs, such as elderly people) (serious concerns about patient privacy and fit with international regulations) |
| 22 | Najla Dar Odeh | Saudi Arabia | Communication | follow cross-infection control protocols, do their best to decide on the emergency cases, be updated on how this pandemic is related to their profession in order to be well oriented and prepared. |
| 23 | Matteo Peditto | Italy | Eexperimental | Transmission Routes, Patient Management, Environments Management, Instruments Management |
| 24 | Luciano José PEREIRA | Brazil | Critical review | keeping only urgent and emergency visits to the dental office. The use of tele dentistry (phone calls, text messages) is a very promising tool to keep contact with the patient without being at risk of infection |
| 25 | Karen Glazer Peres | Australia & USA | Descriptive study | Our students face disruption to their education and career; our professional colleagues will be challenged rebuilding their practices, while staff at all Dental Schools are experiencing various hardships. |
| 26 | GS Vidya | India | Editorial | The biggest challenges faced by health-care professionals are its recognition, providing treatment, and preventing transmission, Wearing a mouth mask, infection control protocol |
| 27 | Yue Sa | China | Descriptive approach | Clinical care for patients with dental emergencies, Additional resources were allocated for facility preparation and management and employee training. Clinical operation protocols were developed, The psychological health and mental wellness of the employees were emphasized. Distance or online education is still under rapid development |
| 28 | Adrian H Shi | Singapore | Review article | Triage of Patients, Standard Precautions and protocols, Personal Protective Equipment , Surgical Masks, Hand hygiene, Biohazard Waste Management, Dental Practice Management Measures |
| 29 | Federico Alcide Villani | Italy | Review article | Precautionary measures should be applied in clinic, using telephone triage and/or  clinic questionnaires, body temperature measurement, usage of personal protective equipment, surface disinfection with ethanol between, use of a rubber dam is essential. |
| 30 | Afisu A. Oladega | Canada | Letter to the editor | Essential and emergency cares, changes in patient volumes, types of procedures  Performed, and the financial impact of the pandemic, Virtual care |
| 31 | VirojWiwanitkit | India | Letter to the editor | For the patients with learning disabilities, a poor collaboration is expected  and the prevention for COVID-19 during dental practice might be very difficult, Only necessary and emergency dental procedures are performed |
| 32 | Movina Wu | Taiwan | Letter to the editor | formulation of a more rigorous and numerical guideline |
| 33 | Liujun Zeng | China | Letter to the editor | Strategic plan for management in oral and maxillofacial surgery (before, during and after operation) |
| 34 | Zhao Zhiguo | China | Descriptive study | Protection of medical staff in department of oral and  maxillofacial surgery and outpatient department, Medical protection for patients with fever, |
| 35 | Matthias Zimmermann | Austria | Review article | Personal protective equipment, Triage and prioritizing of procedures in oral and maxillofacial, Surgery, Aerosol-generating procedures, Outpatient and inpatient care, Precautions in the operating room |
| 36 | Yang Yang | China | Short communication | Health services provision of 48 public tertiary dental hospitals, COVID has strongly affected the dental hospitals` health services. Public sector because of the insurance and the dental resources were crowded. |
| 37 | Sreekanth Kumar Mallineni | USA | Editorial | Role of guidelines and professional judgement, highlight considerations for dentists providing care for children during this pandemic |
| 38 | L. Meng | China | Discovery | Risk of Nosocomial Infection in Dental Settings, Effective Infection Control Protocols, Current Status of Our School and Hospital (Evaluation of Patients., Oral Examination., Treatment of Emergency Cases, Recommendations for Dental Education*)* |
| 39 | Amber Ather | USA | Review article | Patient management and prevention of nosocomial infection: tele screening and triaging, patient evaluation, appropriate use of personal protective equipment |
| 40 | Paul Coulthard | UK | Opinion | concerns about financial consequences, PPE, volunteering and redeployment , moral versus evidence based decision making |
| 41 | Paul S. Casamassimo | USA | Editorial | force emergency patients to seek care at a hospital ED or public training institutions (ethical issue) |
| 42 | B. Ruhin | France | Guidelines | Guidelines and recommendations, Personal Protective Equipment, Handwashing, Management of an asymptomatic patient |
| 43 | Zi-yu GE | China | Descriptive approach | transmission of diseases by aerosols and droplets, Droplets and aerosols in dental setting, precautions in dental setting and emergencies (waiting area, hand hygiene, PPE, rubber dam isolation, mask, removal/filter of contaminated air, shield, mouth rinse, environmental surface disinfection |
| 44 | Gianrico Spagnuolo | Italy | Editorial | Several personal protection measures, use of saliva ejectors, guidelines for patient management and the dental workers safety |
| 45 | Xian Peng | China | Review article | Transmission routs (airborne, contact spread, contaminated surface spread) and infection control (patient evaluation, hand hygine, PPE, Rubber dam isolation, Anti-retraction handpiece, Disinfection of the clinic settings and management of medical waste) |
| 46 | Samy Darwish | UK | General practice | Service provision, Organizing clerical facilities, triage, Managing patients remotely, hand hygiene, Purchase of oral health products, Clinical and social history-taking |
| 47 | Hamid Reza Fallahi | Iran & Korea | Review | there is a high risk of cross-infection, transmission, Infection control, Mouth rinses, Rubber dam isolation, Anti-retraction handpiece, Management of medical waste |
| 48 | Sayna Shamszadeh | Iran | Review | risk of virus transmission can be considered high between patients and dental practitioners., need for strict and tough effective infection control protocols in dental practice is of great importance (hand hygine, PPE, Protective goggles and face shields, mask, gown, ), Surface Disinfection, , Mouth-Rinsing Before Dental Treatment, Removal of Medical/DentalWaste |
| 49 | Imran Farooq | Saudi Arabia | Letter | Dental practice currently is restricted to provision of emergency dental  care whereas, cancelled elective procedures, healthcare workers are also facing financial challenges in this difficult time |
| 50 | Jasmine Marwaha | India &USA | Review | Dental health care professionals are at greater risk, more prone to aerosol infections, follow standard precautions, self-protection, infection control, postpone elective dental procedures, pharmacological management |

**Table 2- Main concerns and sub concerns of dental and oral health workers**

| **Main themes** | **Sub-themes** | **References** |
| --- | --- | --- |
| **Economic concerns** | Economic losses/ financial consequences | 19, 20, 25, 49,50 |
|  | Economic repercussions | 19 |
|  | Economic aid from the government | 19,22 |
|  | Additional costs of control infection | 19,20,21 |
| **Ethical concerns** | Patient privacy | 25 |
|  | Effectiveness of new technologies on population health | 25,26, 27,28 |
|  | Focus on essential and emergency cares | 21,23, 25, 29, 30, 33, 49, 50,51 |
|  | Postpone routine orthodontic appointments | 24 |
|  | Concerns on fitness with international regulations | 26 |
| **Social concerns** | Lack of population`s adequate digital literacy | 26 |
|  | High perceived distress and need for preparedness | 31, 32 |
|  | Feelings of anxiety and fear | 30,22 |
|  | Psychological health and mental wellness of the workers | 33 |
| **Professional concerns** | Impacts of the future of professional | 22,34 |
|  | Immediate impact on dental health care | 32,34 |
|  | Impacts of management of patients with special needs | 35, 36 |
|  | Medico-legal concerns | 32 |
|  | various hardships in rebuilding the practices | 37 |
|  | moral versus evidence based decision making | 20 |

**Table 3- Main coping strategies for oral and dental health workers against COVID-19**

| **Main themes** | **Sub themes** | **References** |
| --- | --- | --- |
| **Patient management strategies** | Guidelines for patient management | 1,30,21,10,39,33,28,32,37,25,41,48 |
|  | Training and education | 21,35,16,33,38 |
|  | Appropriate time management | 24,12, 27 |
|  | Patient screening | 8,27,51,28,38,46,46 |
| **Infection control strategies** | Personal Protective Equipment | 21,24,19,17,18,26,28,29,35,46,20,56,48,41,45,48 |
|  | Environmental sterilization and disinfection | 10,17, 51,26,29,48 |
|  | Rubber dam isolation | 12,17,48,45,47 |
|  | Hand hygiene | 28,48,45,46,48 |
|  | Mouth rinsing and saliva ejection | 41,55,48 |
|  | Removal of Medical/Dental Waste | 28,45,55,48 |
| **Virtual strategies** | Virtual assistant | 29,29,49 |
|  | Telemedicine | 27,42,28 |
|  | Tele dentistry | 29,46 |
